# Editorial: Emerging SARS-COV-2 variants: genomic variations, transmission, pathogenesis, clinical impact and interventions, volume III

**DOI:** 10.3389/fmed.2025.1615467

**Published:** 2025-07-30

**Authors:** Deepak Y. Patil, Pragya D. Yadav

**Affiliations:** ^1^Indian Council of Medical Research-National Institute for One Health, Nagpur, Maharashtra, India; ^2^Maximum Containment Facility, Indian Council of Medical Research-National Institute of Virology, Pune, Maharashtra, India

**Keywords:** emerging SARS-CoV-2 variants, genomic variations, pathogenesis, clinical impact, intervention

Public health strategies and vaccine effectiveness and therapeutic interventions continue to be affected by ongoing evolution of the SARS-CoV-2 through newly detected variants. Various research studies in this Research Topic presents a complete perspective about new SARS-CoV-2 variants by investigating their genomic development and transmission patterns as well as immune evasion processes and clinical manifestations of emerging strains particularly analyzing Omicron subvariants. The editorial combines research insights from these studies into a cohesive presentation which fits within worldwide COVID-19 mitigation strategies.

## A genomic evolution of SARS-CoV-2

A clear comprehension of SARS-CoV-2 genetic evolution is required to track the capacity to evolve and spread. The study by Schnaubelt et al. shows efficiency of self-collected saliva specimens to achieve real-time genomic surveillance in occupational setting. The researchers discovered that Ct value optimization for sequencing referral creates an essential tool for detecting future infectious respiratory pathogen outbreaks (Schnaubelt et al.). This research validates decentralized testing methods as a suitable approach since they provide both accessible examination procedures for other respiratory pathogens alongside scalable and non-invasive testing methods.

Dhankher et al. conducted a detailed investigation of Omicron variant genomic alterations in Madhya Pradesh India by tracking changes from BA.1 to BA.4 while identifying important mutations in spike proteins that affect infectivity and immune evasion. This research demonstrates how SARS-CoV-2 undergoes continuous evolution and need for sustained genomic monitoring of new SARS-CoV-2 variants.

A study by Ravi et al. explored SARS-CoV-2 recombinants that produce distinct mutant patterns leading to decreased virulence and transmission potential in viral evolutionary mechanisms. Their investigation reveals that recombination affects variant fitness together with evolutionary adaptation within populations with strong immune exposure (Ravi et al.). The genomic surveillance report from Weihai, China by Li et al. shown how Chinese COVID-19 policy changes affected virus spread dynamics and evolution. Ayubov et al. discovered new amino acid variations found in SARS-CoV-2 genomes of Uzbek patients revealing details about Delta variant mutations. The researchers demonstrate that monitoring of local genomic sequences continues to be essential for tracking distinct evolutionary patterns of variants (Ayubov et al.). Continued surveillance practice becomes essential according to Potdar et al. through implementing influenza-like illness (ILI) and severe acute respiratory infections (SARI) monitoring within SARS-CoV-2 surveillance systems to establish comprehensive epidemiological investigation.

## Immune escape, pathogenesis, and transmission

The public health consequences of new SARS-CoV-2 sub-lineages depend on their ability to transmit as well as evade immune responses. Barrera et al. demonstrated through their research that BQ.1.1 derived from BA.5 shows higher neutralization escape compared to XBB.1.5 which is derived from BA.2. SARS-CoV-2 shows a naturally adaptive pattern because it successfully achieves both immune evasion mechanisms and transmission capabilities.

Jang et al. studied how increased protection from vaccination affects the reproduction number throughout different variant outbreaks through statistical modeling which demonstrated substantial reduction in viral spreading. The study promotes prompt booster immunizations.

The research by Iversen et al. demonstrates that patients with Delta or Omicron infections exhibited worsening symptoms of dyspnea and cognitive dysfunction along with depression, but vaccination prevented patients from enduring extended sick leaves. The study emphasizes the requirement of specialized rehabilitation programs for long-COVID patients while advocating continuous studies on pathophysiological mechanisms of extended symptoms.

The study by Lin et al., explores the post-Omicron wave resurgence of Influenza A by showing clinical outcome differs among pediatric COVID-19 patients from those with influenza infection The research establishes how easing COVID-19 restrictions allows other respiratory viruses to resurge and importance of continued surveillance. An epidemiological study and laboratory analysis of Omicron infections in Guangzhou presented by Chen J. et al. showed age-related viral load differences and Ct value associations with clinical parameters to enhance understanding of SARS-CoV-2 diagnostic methods.

## Risk factors and clinical outcome associated with severe disease

Clinical assessments must continue to evaluate new SARS-CoV-2 variants in order to understand their impact in different population groups. Research by Liu et al. shows that cancer patients infected with Omicron usually experience mild disease, but specific comorbidities raise the risk of mortality. The research findings confirm that immunocompromised individuals need priority access to booster doses together with targeted antiviral treatment.

Yu et al. demonstrate that cardiovascular disease stands as the major risk element for serious COVID-19 medical complications while C-reactive protein (CRP) and D-dimer levels function as essential measures to forecast mortality outcomes. The research demonstrates the requirement of testing cardiovascular risk profiles for managing COVID-19 patients particularly within elderly groups (Yu et al.)

The study carried out by Arunthai et al. demonstrated that patients with elevated CRP levels presented greater chances of ICU hospitalization and acute kidney injury and mortality. The results demonstrated that CRP serves as an important signifier of COVID-19 clinical outcomes during management (Arunthai et al.)

The study by Tsai et al. investigates association of immune marker associated with disease severity in multisystem inflammatory syndrome in children (MIS-C) through analyses of cytokines and miRNAs. Bacterial superinfections among hospitalized children with COVID-19 has been studied by Lai et al. who determined that abdominal pain and diarrhea and neurologic comorbidities serve as risk factors for bacterial infections.

Birhanu et al. evaluated mortality predictors among Ethiopian COVID-19 patients using a systematic review and meta-analysis approach which determined chronic kidney failure, diabetes, hypertension, smoking and HIV infection as risk factors leading to fatal outcomes. Clinical management strategies must particularly target high-risk individuals in an attempt to reduce mortality rates. Epidemiological modeling techniques from Ma et al. monitor SARS-CoV-2 transmission within Shanxi Province to demonstrate how effective public health interventions helps to reduce infections.

## Insights of immune response to SARS-CoV-2 variants

A thorough examination into immunological insights of evolving SARS-CoV-2 variant becomes essential for creating and optimizing preventive measures and vaccination programs. The research by Soni et al. uses single-cell genomics methods to examine COVID-19 patient T-cell immune responses in order to create new directions for immunotherapy (Soni et al.). Khare et al. reveal that metal ion homeostasis disruptions function as a new mechanism to affect CD8+ T cell capability during SARS-CoV-2 infection thus expanding knowledge of unconventional immune pathways.

The study by Chen X. et al. examines IgG levels to determine their relation with symptomatic Omicron infections in children while suggesting protective immunity thresholds. The research outcomes corroborate with other studies that proves booster vaccines help maintain defense against variant evolution. Hunter et al. developed SCENTinel^®^ which demonstrates superior performance than symptom checklists in SARS-CoV-2 detection specifically during the Delta wave and enables population-wide screenings through its rapid olfactory assessment technology.

## Conclusion

The current situation with SARS-CoV-2 variant emergence demands collaborative approach combining genomic surveillance methods and immunological research and clinical evaluation and public health implementation. This Research Topic includes a comprehensive collection of research articles that describe major advances in the research on viral evolution and transmission and their clinical significance. These findings will help in developing preparedness strategies and optimizing interventions for evolving SARS-CoV-2 variants in future.

